# Supplementary material for: The Role of Posterior Neural Plate-Derived Presomitic Mesoderm (PSM) in Trunk and Tail Muscle Formation and Axis Elongation
Source: Cells. 2023 May 4;12(9):1313. doi: 10.3390/cells12091313 (PMC10177618; doi:10.3390/cells12091313)
Supplement: Supplementary file 1 [file cells-12-01313-s001.zip › cells-2330103-supplementary.pdf]

Supplementary Figure legends

Figure S1. Tail measurements and measurements of PSM tissue in 3D reconstructions. **(a)** Schematic of a stage 30 embryo, visualizing the transversal and horizontal ledger lines for tail length measurements. **(b)** Calculation of the length of mesodermal (PSM) tissue (GFP^+, grey), here visible next to the neural tube ($Sox2^+$, red) **(c)** Calculation of PSM tissue height in different anteroposterior regions; **(d)** Calculation of tissue width in different anteroposterior regions. The length, height and width of GFP^+ mesoderm was measured using the Fiji 3D Viewer Plugin [1]. For each feature, feature specific reference points (yellow dots) were selected and Euclidian distances were calculated between them. PSM: presomitic mesoderm; NT: neural tube.

Figure S2. Manual cell segmentation and ellipsoid fitting: (**a**) Individual single lifeact-GFP^+ cells in a z-stack were **(b)** manually segmented, and **(c)** an ellipsoid was mathematically fitted into the segment for each cell. **(d)** Then poles of the cell were modelled by points A and B. The vector between both points represents the orientation of the cell. The centre is labelled as C.

Figure S3. Calculation of individual cell distances **(a-b)** and closest neighbour analysis **(c-d)**: **(a)** Individual cells from a small lifeact-GFP-labelled group were segmented manually. **(b)** Ellipsoids were fitted and pairwise distances between their central points calculated. **(c)** Segmented GFP^+ cells were filtered and grouped into different mesodermal regions according to their location. Spheres represent mesodermal regions within GFP^+ PSM. Each sphere is defined by its central point S and a radius r (not shown). **(d)** Distances between all GFP^+ cells are represented by black lines; 5 shortest distances per cell (red lines) are determined to calculate correlates of cell density in this region. (nt: neural tube; ant. PSM: anterior presomitic mesoderm; post. PSM: posterior presomitic mesoderm; PZ: posterior zone; S1-S3: somites 1 to 3).

Figure S4. Measurement of cell orientation within mesodermal regions: **(a)** 80 μm thick transverse tissue sections were cut from the posterior end of a stage 22 embryo to image single lifeact-GFP^+ cells. Body axis of the embryo in the tissue section are depicted schematically (A: anterior, P: posterior, L: lateral, M: medial, D: dorsal, V: ventral). According to the dimensions of the transverse tissue section (red dashed line), the calculated anterior-posterior axis would be in an orthogonal straight line (green dashed line). The real anterior-posterior axis is curved and follows the neural tube. To find the correct cell orientation within the tissue section, orientation vectors in the tissue section must be corrected according to the true AP axis in the embryo. **(b)** Visualization of uncorrected cell orientation measurement in tissue slides. Segmented cells were represented by the vector $\overrightarrow{AB}$ between the points A and B at the poles of an ellipsoid (green) fitted into the cell. The angle of intersection φ_{xy}, φ_{yz} and φ_{xz} between $\overrightarrow{AB}$ and the normal vectors $\overrightarrow{n_{xy}}$, $\overrightarrow{n_{xz}}$ and $\overrightarrow{n_{yz}}$ of the transversal (E_{xy}), sagittal (E_{yz}) and longitudinal (E_{xz}) plane determine the orientation of the cell in the coordinate system (grey). **(c)** Visualization of corrected cell orientation measurement according to AP axis in the embryo. To match cells in the tissue section to the situation in the embryos, the anteroposterior axis was modelled as a tangent at the point of the performed transversal tissue sections. The transversal plane at this part of the embryo is orthogonal to the tangent. The corrected transversal plane differs from the cut transversal plane by the angle (α). The coordinate system generated by the image dimensions (grey) was transformed according to the measured angle (α) in each image set (purple). The transformation of the coordinate system generated new normal vectors of the coordinate planes ($\overrightarrow{n_{\alpha xz}}$) and ($\overrightarrow{n_{\alpha xy}}$) (purple). These corrected normal vectors were used to calculate the orientation of cells according to their position within the embryo. **(d)** Formulas used to transform normal vectors of the coordinate planes in the tissue section $\overrightarrow{n_{xy}}$ and $\overrightarrow{n_{xz}}$ into corrected normal vectors ($\overrightarrow{n_{\alpha xy}}$) and ($\overrightarrow{n_{\alpha xz}}$) rotated by angle ($\alpha$).

Figure S5. DiI-labelling of lateral plate mesoderm/endoderm in the lateral trunk. **(a)** Schematic showing focal DiI injections into the lateral trunk at stage 22 and expansions of the DiI-labelled sites until stage 40. **(b)** Determination of DiI injection sites at various stages after injection at stage 22. D_{rel} indicates relative distance to anterior body border. **(c)** Representative images of embryos showing distribution of DiI label from the time of injection (stage 22) to a larval stage (stage 40). Number of embryos injected per site: n=6 (each) in mid-trunk and trunk-tail transition zone, and n=4 in the tail bud area; endo: endoderm; myo: myotome; nt: neural tube.

Figure S6. DiI-labelling of ventral endoderm in the ventral trunk. The outer layer of the endoderm (future mucosa) and occasionally yolk platelets of the future gut are labelled. **(a)** Schematic showing focal DiI injections into the ventral trunk at stage 22 and expansion of the DiI-labelled sites until stage 40. **(b)** Determination of DiI locations at various stages after injection at stage 22. D_{rel} indicates relative distance to anterior body border. **(c)** Representative images of embryos showing distribution of DiI label from the time of injection (stage 22) to a larval stage (stage 40). Cells labelled in the trunk-tail-transition zone were later not only found in the endoderm (hindgut)

but also in myotomes and somite-derived ventral fin mesenchyme at stages 35 and 40 (due to co-labelling of endoderm and PSM because of close tissue proximity in the tailbud). Number of embryos injected per site: n=6 embryos in the head-trunk transition zone, n=5 in the mid-trunk, and n=8 in the trunk-tail-transition zone.

Figure S7. Orthotopic grafting of GFP$^+$ labelled epidermis or paraxial mesoderm. **(a)** D_{rel}, calculation of the relative location of transplanted GFP$^+$ tissue. **(b)** Locations of transplanted GFP$^+$ cells at different embryonic positions based on relative distances to anterior body border at stage 40; **(c-j)** Representative images of embryos with GFP$^+$ grafts transplanted at stage 22 (schematics) and their corresponding locations at stage 40. Grafting details: GFP$^+$ labelled epidermis **(c, e, g, i)** or paraxial mesoderm **(d, f, h, j)** grafted from stage 22 GFP$^+$ donors to white (*d/d*) hosts and followed until stage 40; locations of tissue grafts were in the dorsal mid-trunk **(c, d)**, trunk-tail-transition zone **(e, f)**, tail bud **(g, h)** and tail tip **(i,j)**. White or green arrows point to the centre of the GFP-labelled area in epidermal and mesodermal grafts, respectively. Green colour in the trunk of the embryo **(j)** is due to autofluorescence of the yolk in the endoderm. Number of transplanted embryos: n=2-4 per group.

Figure S8. The image shows a single plane of a 3D reconstruction of a z-stack through a vibratome section of the PSM of a stage 22 embryo. DNA was stained with propidium iodide. The magenta dots mark the opposing poles of the nucleus (labelled A and B) connected by a solid white line, which indicates the orientation of cell division. The division plane (dashed white line) is perpendicular to this line. The cell nuclei of surrounding cells are not in mitosis.

Figure S9. Orientation of PSM cells in the tissue **(a-c)**, and number and length of their filopodia **(d-e)**. **(a)** Experimental design: a small part of the medial posterior NP was transplanted from a stage 15 lifeact-GFP$^+$ donor to a *d/d* host. Labelled cells were analyzed at stages 22, 28 and 30 in the indicated locations. **(b)** Pie charts of calculated cellular orientations. Cell orientations were significantly non-randomly distributed (Chi-square X^2 statistical test, $p < 0.0001$). Number of cells analyzed (per region): n=71 (PZ), 38 (post. PSM), 38 (med. PSM), 55 (ant. PSM) and 112 (somites). Cells from n=3-4 embryos were analysed. **(c)** Schematic summary of results on cell shape and orientation in different parts of the tail PSM. Orientation of cells in the reference embryo is colour-coded and depicted in a coordinate diagram. **(d)** Number of filopodia per cell per region depicted as percentage of cells with a given number of filopodia. Number of cells and embryos analyzed (per region) as in **(b)**. **(e)** Average length of the filopodia per region; statistical differences are shown (1-way ANOVA: * p<0.05,** p<0.001, *** p<0.0001; relations not indicated were not significantly different). Number of filopodia analyzed (per region): n=97 (PZ), 51 (post.PSM), 62 (med. PSM), 247 (ant. PSM) and 116 (somites). Cells from n=3-4 embryos were analysed.

Figure S10. Influence of the removal of tail epidermis on PSM and tail morphology. **(a)** Experimental design: the left half of the posterior NP was transplanted from a stage 15 GFP$^+$ donor to a *d/d* host. At stage 22, the epidermis was removed between the last somite and the tail tip from the left, right or both sides of the host embryo (dark grey). The tail length and bending were then measured at stage 30. **(b)** Examples of stage 30 embryos (imaged from the left side) with the intact epidermis (control) and after removing the tail epidermis at stage 22 from the left side, right side or both left and right side. **(c)** 3D reconstructions of whole-mount immunostained embryos against GFP and Sox2 after epidermis removal, optical tissue clearing and microscopic imaging. **(d)** Height (dorsoventral extension) of the GFP$^+$ PSM tissue. **(e)** Length (anterior-posterior extension) of the GFP$^+$ PSM tissue. **(f)** Width (mediolateral extension) of the GFP$^+$ PSM tissue. **(g)** GFP$^+$ tissue volume; no significant differences were found (1-way ANOVA). Methods of measurements in d-g are shown in Figure S1. Numbers of analysed embryos per group: n=5 (control), 8 (epidermis removed from the left side), 8 (epidermis removed from the right side) and 9 (epidermis removed from the left-right side). PZ: posterior zone; NT: neural tube.

Figure S11. Distances of closest neighbours among PSM cells after epidermis removal. **(a)** Experimental design: The left half of the posterior NP was transplanted from a stage 15 GFP$^+$ donor to a *d/d* host. At stage 22, the epidermis was removed between the last somite and the tail tip from the left, right or both sides of the embryo (dark grey). Cell distances were analysed at stage 30. **(b-g)** Averages of the distances to the five closest neighbours of each analysed cell were calculated per region as a measure for cellular density. Significant differences between control and epidermis removed embryos are indicated (1-way ANOVA: * p<0.005, *** p<0.0001). Number of cells analysed per region (condition): PZ – n=233 (control), 124 (left side), 272 (right side) and 118 (left-right side), post. PSM – n=200 (control), 200 (left side), 249 (right side) and 137 (left-right side), ant. PSM – n=65 (control), 51 (left side), 70 (right side) and 22 (left-right side), S1 – n=65 (control), 63 (left side), 65 (right side) and 38 (left-right side), S2 – n=71(control), 64 (left side), 59 (right side) and 20 (left-right side), S3 – n=53 (control), 53 (left side), 28 (rightside) and 22 (left-right side). Number of embryos analysed per condition: n=4 (control), 6 (left side), 6 (right side) and 5 (left-right side).

Figure S12. Rescue experiments after epidermis removal. **(a)** Experimental design: The epidermis was removed between the last somite and the tail tip from the left side of the embryo (dark grey) at stage 22. Following epidermis removal embryos were embedded in 3% methylcellulose or 1% agarose with or without fibronectin. Control embryos (without epidermis removal) were also embedded. The embryos were allowed to develop until stage 30 and analyzed for the presence of defects in tail development. **(b)** Example images of stage 30 control and epidermis-removed embryos embedded in 3% methylcellulose or 1% agarose with or without fibronectin. Black arrows mark the trunk-tail transition zone. Control embryos show a normal morphology while epidermis-removed embryos embedded in 3% methylcellulose or 1% agarose without fibronectin as well as in 3% methylcellulose with fibronectin show shortened tails with no ventral bending. Only the epidermis-removed embryo embedded in 1% agarose with fibronectin shows a rescued tail morphology.

Supplementary Materials and Methods

1. Animals

Eggs of white mutant (*d/d*) (host) and transgenic GFP-expressing white mutant (*d/d*) (donor) females (*CAGGs:EGFP*; designated as GFP^+) of the Mexican axolotl (*Ambystoma mexicanum*) were obtained from the axolotl-facility at the Center for Regenerative Therapies (CRTD), TU Dresden. Eggs of lifeact-GFP transgenic axolotls (*tgSceI(CAGGs:lifeAct)*^{PMX}) were kindly provided by Prayag Murawala, Ph.D (MDI Biological Laboratory, USA). For development, eggs were kept in tap water at room temperature (RT) or 11°C. Before experiments embryos were transferred into cold (11°C) sterile 1x Steinberg's [2] solution supplemented with 1% penicillin-streptomycin (Biochrom) and 3 mg/ml Ciprobay (Bayer AG) in plastic dishes coated with sterile 2% agar dissolved in tap water. Their jelly coat was removed with forceps, then embryos were screened for transgenesis under UV light and sorted into GFP^+ and GFP^-. During experiments, embryos were kept in agar dishes filled with 1x Steinberg`s solution at RT or at 11°C. Before fixation with 4% paraformaldehyde (PFA; Cart Roth GmbH) in 0.1 M phosphate buffer, embryos were anaesthetised with 0.03% benzocaine for 20 min.

2. DiI-labelling of tissues

Experiments involving DiI-labelling of tissues were used to label only small groups of cells (ca. 5-10) in contrast to tissue grafting of GFP^+ transplants into white (*d/d*) hosts, which comprised large cell numbers (hundreds or thousands). For DiI injections, embryos (stage 22) were placed in a deepening of an agar-coated dish filled with 1x Steinberg's solution. Tailbuds at this stage are most suitable for our research because the prospective head, trunk and tail segments can be discriminated for the first time and the occurrence and degree of displacement of a labeled tissue in an anterior or posterior direction can be studied. For injections, a 3.5'' capillary with a sharpened mouth was filled with DiI (Invitrogen) solution in 1 µm/µl in DMSO (Sigma Aldrich). With the help of a microinjector (Drummond, Nanoject II) DiI (5 nl; 1 µg/µl) was injected under a stereomicroscope into defined positions in the developing embryo. After the injections, embryos were kept separately in an agar-coated dish at RT or 11°C and imaged daily until stage 40. Locations of DiI-labelled cells were examined and measured at stages 22, 25, 30, 35 and 40 and summarised graphically. To assess the contribution of injected cells to axis elongation, locations of the DiI-labelled areas along the AP axis at each developmental stage were normalized to the entire body length. For each injected embryo the 'relocation's relative distance' (d_{rel}=a/b) was calculated, where a is the distance between the anterior-most end of the animal and the center of the labelled region in parallel to the ventral body lining of the embryo, and b is the total length of the animal. Length measurement in axolotl embryos (stages 22-40) was performed on images generated using a stereomicroscope (Zeiss).

3. Homotopic grafting of GFP^+ tissues

Before the operation, donor and host embryos were transferred to an agar-coated plastic dish containing 4x Steinberg's solution (11°C) which facilitates tissue separation. To immobilize the embryos, they were placed next to each other in a small groove in the agar. Then the vitelline membranes were removed with forceps under a stereomicroscope. Subsequently, the GFP^+ tissue to be grafted was cut out from the GFP^+ donor using tungsten needles and transplanted into a white mutant (*d/d*) host. After transplantation, operated hosts were transferred into a new dish filled with 1x Steinberg's solution and kept there for wound healing and further development. Tissue grafts were covered with a small sterilised glass plate to hold the tissue in place. After 10-15 minutes, the glass plate was carefully removed. Homotypic tissue transplants were grafted between embryos of the same age (stage-matched isochronic grafts). The embryos were then allowed to develop until an appropriate stage. The following types of transplantation experiments were performed:

3.1 Transplants of the entire posterior NP at stage 15 (area ca. 300 x 1000 µm). The posterior NP was transplanted from ubiquitously GFP-expressing or lifeact-GFP white mutant (*d/d*) donors to white mutant (*d/d*) hosts to label the entire prospective presomitic mesoderm (PSM) of posterior trunk and tail of the embryo. Chimeric embryos were then used for either cutting transverse or median tissue sections (through the GFP^+ labelled tail region) or whole mount experiments for 3D-reconstructions and measurements of the GFP^+ tail mesoderm.

3.2 Transplants of the left posterior NP at stage 15 (area ca. 300 x 500 μm). Tissue was grafted from GFP$^+$ donors to white (*d/d*) mutant hosts. These transplants were used to:
  - investigate migration and extension of prospective PSM area with regards to midline-crossing of PSM cells.
  - study the effects of epidermis removal at stage 22 on the behaviour of PSM tissue. Embryos treated in this way were allowed to develop to stage 30. Then the dorsolateral epidermis between the last formed trunk somite and the tail tip on the left, right and on both sides of tailbuds was removed.

3.3 Transplants of small posterior NP areas at stage 15 (ca. 20 cells) in a median, paramedian or lateral position were performed for studying:
  - distribution of small cell groups transplanted from a median, paramedian or lateral area of the GFP$^+$ left posterior NP into white mutant donors. Their distribution was analyzed at stages 28, 30 and 35.
  - shape and orientation of individual mesodermal cells. Only median grafts from lifeact-GFP$^+$ donors were performed and analyzed at stages 28, 30 and 35.

3.4 Transplants of small epidermis and paraxial mesoderm areas at stage 22 (area ca 1 x 1 mm). Four positions of the transplants were chosen in the dorsolateral trunk and tail: midtrunk, trunk-tail transition site, tailbud and tail tip. For epidermis transplants, host (*d/d*) surface epidermis was replaced with GFP$^+$ epidermis of the donor. For paraxial mesoderm transplants (grafts of somites or PSM depending on the axial position) the host epidermis over the graft site was lifted, the host mesoderm portion removed and the GFP$^+$ tissue implanted. Host epidermis was then gently stretched back over the wound. These experiments were used to distinguish between the ability of epidermis and mesoderm to expand along the body. Such a distinction could not be achieved with DiI labelling that hit only mesoderm. The calculation of 'relocation's relative distance' (d_{rel}=a/b) was done exactly as for the DiI-labelling experiments (2).

4. Histological stainings

4.1 Vibratome sections:

Embryos were fixed with 4% PFA in 0.1 M phosphate buffer at 4°C overnight, postfixed with methanol/DMSO [3] and stored in methanol at -20°C. Methanol-fixed samples were rehydrated via a descending series of methanol (80% / 50% / 30%) in PBS and then washed with PBS for 30 min. Because actin filaments are sensitive to methanol, animals containing lifeact-GFP transplants and animals used for staining of actin filaments were fixed only with PFA. These samples were washed 3 times with PBS for 30 min before further use. Fixed specimens were embedded in 4% agar dissolved in tap water in the desired orientation. Transverse vibratome sections (80 μm thick) were cut through the embryos starting from their caudal ends. Sections were collected individually in single wells of a 24-well plate filled with PBX (1x PBS with 0.05% Triton X-100). Sections were then blocked in 10% normal goat serum in PBX for 1 h at RT. Blocking was followed by incubation with primary antibodies in 10% normal goat serum in PBX overnight at 4°C. Sections were then washed with PBX 3 times for 30 minutes and incubated with secondary antibodies in 10% normal goat serum in PBX for 1 h at RT. Then, sections were washed again with PBX 3 times for 30 minutes. For optical tissue clearing, sections were incubated with primary antibodies for 3 days at RT and with secondary antibodies for 2 days. After staining, sections were transferred individually to a glass slide and covered with mounting medium (FluoroMount) and a coverslip. During mounting all sections were oriented with the anterior side facing the coverslip under a stereomicroscope. Images were taken with a Zeiss OBSERVER Z.1 wide-field microscope with apotome using EC Plan-Neofluar 20x/0.5 or Plan Apochromat 20x/0.8 objective. Antibodies were used at the following dilutions: 1) primary antibodies: chicken α-GFP (Abcam, ab13970) 1:1000, rabbit α-Sox2 [4] 1:500; 2) secondary antibodies: AffiniPure F(ab')$_2$ Fragment donkey α-chicken IgY (IgG) (H+L) Alexa Fluor 488 (tissue sections) (Jackson ImmunoResearch, 703-546-155), AffiniPure F(ab')$_2$ Fragment donkey α-chicken IgY (IgG) (H+L) Alexa Fluor 647 (whole mount staining) (Jackson ImmunoResearch, 703-606-155), goat α-rabbit IgG F(ab')2 Alexa Fluor 555 (Cell Signaling Tech., #4413). Nuclear staining was done with DAPI (4',6-diamidin-2-phenylindol; Thermo Fisher Scientific) at 1:1000 dilution.

4.2 Plastic sections:

For plastic sections, first the whole embryos were immunolabelled and embedded into Technovit 7100 resin (Heraeus-Kulzer, Wehrheim, Germany) as described [5], [6]. Samples were fixed in 4% buffered paraformaldehyde (PFA) and methanol/DMSO [3], rehydrated in a graded series of methanol and PBS, and blocked with 20% normal goat serum (NGS) in PBS before staining with primary antibodies against actin (mouse α-actin: CLT9001, Torrey Pines, 1:200), and β-catenin (rabbit α-β-catenin: P14L, 1:50) [7], followed by secondary antibodies conjugated to Alexa555 and Alexa488, respectively. The samples were postfixed in 4% PFA, dehydrated in a graded series of ethanol and infiltrated and embedded in Technovit 7100. Serial sections (2 μm) were collected and stained with DAPI before mounting with Mowiol/DABCO. Images were taken on a Keyence Biozero 8000 fluorescence microscope.

4.3 Toluidine blue staining:

Embryo fixation, embedding and sectioning was done as for immunostaining of plastic section. Alternating sections were stained with 1% toluidine blue/0.5% borax. Images were taken on a Keyence Biozero 8000 fluorescence microscope.

4.4 Whole-mounts:

The whole-mount staining for 3D reconstructions was adapted from an existing protocol [5]. Embryos fixed with PFA/Dent`s and stored in methanol were rehydrated and washed as described in *2.4*. As the pigmented epidermis inhibits penetration of the laser light during imaging, pigments were bleached according to the protocol adapted from [8]. Briefly, samples were treated with 0.5% KOH/ 3% H_2O_2 for 1-1.5 h at RT until the embryos appeared yellowish-white. The alkaline bleaching solution was removed by extensive washing with PBS 3 times for at least 30 min. Afterwards, the embryos were incubated with primary antibodies in 10% normal goat serum in PBS for 3 days at RT in closed cryotubes and then washed with PBX in a series of 30 min, 1 h and 2 h washes. Incubation with the secondary antibody was performed for 2 days at RT followed by a series of 30 min, 1 h and 2 h washes with PBX.

5. Optical tissue clearing

5.1 Ethyl cinnamate-based optical tissue clearing:

For the optical clearing of complete embryos, a protocol adapted from Masselink and colleagues was used [9]. The embryos were bleached and immunostained as described (*2.4*). Embryos were then dehydrated for at least 2 h in an ascending series of 1-propanol (Merck)/PBS dilutions (30% / 50% / 80%) pH = 9 with trimethylamine (to adjust the pH; TGI) for better preservation of fluorescent signals. Afterwards the samples were incubated in 100% 1-propanol and then transferred to 100% ethyl cinnamate (ECi, 3-phenyl-2-propenoic acid ethyl ester; Merck) for 2 h to adjust the refractive index of the tissue (clearing step). Microscopic imaging was performed in the clearing solution with a Zeiss LSM 880 microscope. The images were acquired with an EC Plan-Neofluar 10x/0.3 objective and a DPSS 561 nm and a HeNe 594 nm laser.

5.2 SeeDB optical tissue clearing:

The SeeDB clearing protocol was adapted from [10] for 80 μm thick vibratome tissue sections (*2.4*). After immunostaining, tissue sections were fixed again with 4% PFA in 0.1 M phosphate buffer for 1 h at RT, followed by two 10-minute PBS washing steps. Samples were then incubated in a series of 20% / 40% / 60% fructose (Applichem GmbH)/PBS solutions with 100 μl/20 ml α-thioglycerol for 2 h each, followed by overnight incubation in 80% fructose/PBS. Sections were then incubated in 100% fructose/H_2O for 1-2 days and SeeDB/H_2O solution for 1-2 days. All incubations were performed in a 24-well plate on a rocking platform at RT and protected from light. Sections were mounted in SeeDB solution on a glass slide with the anterior side facing upwards. Imaging was performed with a Zeiss LSM 510 laser scanning microscope with an EC Plan-Neofluar 40x/1.3 Oil DIC M27 objective and 488 nm Ar laser.

6. Image generation and processing

Optically cleared whole embryos or tissue sections were imaged as z-stacks with a confocal microscope. Tissue sections were imaged with a Zeiss LSM 510 microscope in 0.1 x 0.1 x 0.4 μm and the whole embryos with a Zeiss LSM 880 in 0.8 x 0.8 x 7.3 μm resolution/voxel size. Images were visualized with ZEN 2012 blue or ZEN 2012 SP1 black software (Zeiss). The 3D reconstructions were either produced with Fiji [11] or Arivis Vision 4D 3.3.0 (Arivis AG) software. Fiji was chosen for manual cell segmentation, filopodia segmentation and GFP^+ tissue volume calculation using 3DManager [12] and 3D Viewer Plugin [1]. Arivis was used for automatic cell segmentation of GFP^+ cells and the generation of custom adjusted transversal and sagittal planes, fitting to the anatomy of individual embryos.

7. Analysis of embryonic features

Statistical analysis and data visualization of embryonic features was done with GraphPad Prism 5.03 (GraphPad Software) and Inkscape 0.92.

7.1 Quantification of embryonic and tissue dimensions:

Length measurement in axolotl embryos was performed on images generated using a stereomicroscope (Zeiss). To measure the length of the tail at stage 30 geometrical ledger lines marking morphological reference points were introduced to normalize the measurements between different embryos (Figure S1a). First, a horizontal line linking the ventral neck groove and the ventral groove at the trunk-tail transition zone was drawn. Then, a transversal line through the cloaca, orthogonal to the horizontal line was drawn to mark the border between trunk and tail. The length of the tail was then measured as a direct line (tail length line) linking the transversal line to the caudal-most end of the embryonic tail, parallel to the dorsal lining. The angle between the tail length line and the transversal line was also measured, to indicate the degree of dorsoventral tail bending. Lateral bending of the tail was measured in embryos imaged from the ventral side in relation to the midline. The length of the left lateral side from the gill bulge to the tail tip was divided by the length of the right side to generate a coefficient representing the straightness of the tail. These parameters were used to assess defects in tail morphogenesis.

To measure the length, height and width of the GFP^+ mesoderm tissue on 3D reconstructions the Fiji 3D Viewer Plugin was used [1]. For length measurement reference points were chosen at the anterior- and posterior-most ends of the tissue and the sites of physiological tissue bending (Figure S1b). The coordinates of each point P (P_x, P_y, P_z) were used to calculate distances between consecutive points from the rostral to caudal end of the tissue. The sum of these distances represents total tissue length. Height and width were calculated by choosing pairs of reference points on opposite edges of the labelled tissue (on dorsoventral or mediolateral axis for height and width, respectively) and calculating the distances between each pair of points (Figure S1c-d). The pairs of points were chosen in different regions (PZ, posterior, medial and anterior PSM, somites: S0 and S1) and height and width were calculated for each region separately.

7.2 Manual cell segmentation:

Individual mesodermal cells within the tail were analysed for different cellular parameters. To measure these parameters, vibratome sections through embryos containing small median posterior neural fold grafts from lifeact-GFP transgenic embryos (*2.3.3.*) were imaged as a series of z-stacks (Figure S2a). For single cell analysis, all cells which were visually distinguishable in all dimensions from other GFP-labelled cells and contained a nucleus in the imaged z-stack were manually segmented with the Fiji Plugin Segmentation editor (Figure S2b). Thereby, cell borders of each cell were manually outlined in all images of a z-stack. The segmented cells were then used for ellipsoid-fitting and calculation of cellular parameters.

7.3 Automated cell segmentation:

Automatic segmentation of GFP^+ cells in whole-mount immunostained embryos was performed in 3D with Arivis software. Cells were identified based on the fluorescent signals (threshold = 20) in a set diameter of 20 μm with a split sensitivity of 96%. The resulting segments were filtered for a volume of at least 100 μm^3 to exclude small particles and staining artefacts. From each segmented cell, the coordinates of the geometrical center were used for further analysis.

8. Quantification of cellular parameters

Statistical analysis and data visualization of cellular parameters was done with GraphPad Prism 5.03 (GraphPad Software) and Inkscape 0.92.

8.1 Cell shape:

The 3D shape of a cell was determined by mathematically fitting an ellipsoid into the segmented cell with the 3D ellipsoid fitting function 3D ImageJ suite plugin for Fiji (Figure S2c). The ellipsoid was then used as a simplified representation of the cell, and the ratio of the length of the longest axis divided by the length of the shortest axis defined cell shape (Figure S2d). An aspect ratio of 1 corresponds to a perfect sphere, and a higher ratio describes more elongated structures (<3 oval, >3 elongated). The mean coefficient was calculated as an average aspect ratio for all analysed cells in a given region.

8.2 Cell and PSM tissue volume:

The volume of manually segmented cells or the entire GFP$^+$ tissue was calculated using the Fiji 3D manager plugin [12]. The volume of segmented lifeact-GFP$^+$ cells could be calculated directly without any further processing. The image sets of the GFP$^+$ tissue were smoothed beforehand using a 3D Gaussian filter. Smoothed images were segmented and tissue volume calculated.

8.3 Cellular distances of labelled small cell groups:

The coordinates of the central point of the ellipsoid defined the position of a cell in space (Figure S3a). These coordinates were used to calculate the Euclidian distances between each pair of lifeact-GFP$^+$ cells (Figure S3b). Distances were only calculated between cells located in the same mesodermal region, the same tissue section and captured on the same microscopic image. Cell distances were determined as a measure of cell group cohesion.

8.4 Closest neighbour analysis:

The closest neighbour analysis was used to infer cell density. For these calculations, 3D GFP$^+$ mesodermal tissue image sets were used, and individual GFP$^+$ cells were automatically segmented. The resulting dataset of geometrical coordinates of each cell ($C \in \mathbb{R}^3$) was filtered according to the position of the cell within the mesoderm (Figure S3c). Mesodermal regions were identified by the tissue morphology in the 3D reconstruction of the whole tissue and defined by a 3D sphere with a central point $S \in \mathbb{R}^3$ and radius r. The central points were manually determined for each mesodermal region in each embryo. The radius was set to 100 µm for the PZ and posterior PSM. The measured height and width of the anterior PSM and somites led to a radius of 50 µm in these regions. A cell was assigned to a mesodermal region if it was located within the corresponding sphere. That was true if the Euclidian distance from the cellular geometrical central point to the central point of the sphere was smaller than the radius of the sphere ($\overrightarrow{CS} < r$). The filtering approach generated different groups of cells, according to their affiliation with a specific mesodermal region. The distances between all cells of such a group were calculated (Figure S3d). The five shortest distances between a cell and its surrounding cells were considered. Those were plotted and compared between different groups.

8.5 Cell orientation:

The orientation of a cell was represented by the orientation of the fitted ellipsoid within a 3D coordinate system representing a tissue section (Figure S4a). The coordinates of the two poles of the ellipsoids (A, B) were used to determine the vector ($\overrightarrow{AB}$) and its orientation, representing the orientation of the cell (Figure S4b). To be able to evaluate the orientation of ($\overrightarrow{AB}$) in space, angles (φ_i) with respect to the normal vectors of each coordinate plane ($\overrightarrow{n_{yz}}$, $\overrightarrow{n_{xz}}$and ($\overrightarrow{n_{xy}}$) were determined. If $\overrightarrow{AB}$ was parallel to one of the normal vectors $\vec{n}$ or intersected in a minimal angle of $\varphi \approx 0°$, the corresponding cell was considered to be oriented like this normal vector. Here, the general orientation of cells was of interest. Therefore, an angle of 0-45° between $\overrightarrow{AB}$ and $\vec{n}$ was considered as the same orientation of $\overrightarrow{AB}$ and $\vec{n}$. This meant the cell was orthogonally oriented to the plane. For example, a dorso-ventrally oriented cell would have $0° \leq \varphi_{xz} \leq 45°$, but $\varphi_{yz} \approx 90°$ and $\varphi_{yz} \approx 90°$. Thus, $\overrightarrow{AB}$ would be orthogonal to the

longitudinal plane. Respectively, anterior-posteriorly oriented cells had a $\varphi_{xy} \leq 45°$ and were orthogonal to the transversal plane. Similarly, mediolaterally oriented cells had a $\varphi_{yz} \leq 45°$ and were orthogonal to the sagittal plane.

$$\cos(\varphi) = \frac{\overrightarrow{AB} * \vec{n}}{|\overrightarrow{AB}| * |\vec{n}|}$$

Embryos were cut transversally. Thus, the resulting anteroposterior axis would be in a direct line from head to tail. According to the anatomy of the embryos, the real anterior-posterior axis follows the shape of the neural tube, which is identical to the bending of the back, respectively (Figure S4a). This bending was measured by the angle α between the artificial section plane and the real transversal plane, which is the orthogonal axis of the tangent of the back of the embryo at the approximate cutting region. Therefore, a rotation around the x-axis by α transformed the normal vectors $\overrightarrow{n_{xz}}$ and $\overrightarrow{n_{xy}}$ into $\overrightarrow{n_{\alpha xz}}$ and $\overrightarrow{n_{\alpha xy}}$ and corrected all calculated (φ_i) to match the real situation in the embryo (Figure S4c-d).

8.6 Number and length of filopodia:

Lifeact-GFP+ cells with filopodia were fixed with 4% PFA (no methanol). Fixation might cause a shrinkage and deviation from the normal orientation, however, most filopodia were still straight after fixation without severe disorientations or destructions. Only such filopodia were included in the measurements. An individual filopodium was characterised by the coordinates of its root $R \in \mathbb{R}^3$ and its tip $T \in \mathbb{R}^3$. Each lifeact-GFP+ cell was screened for the occurrence of filopodia. If a filopodium was found, the coordinates of R and T were extracted from z-stacks with Fiji. The length of a filopodium was calculated as the Euclidian distance between T and R.

8.7 Mitotic spindle orientation:

Tissue sections were stained with propidium iodide to label DNA in the cell nucleus to identify the cells in mitosis and distinguish mitotic phases. The z-stack images were manually analysed for mitotic cells. Mitotic spindles of cells in the metaphase were used for the analysis of orientation of cell division. The coordinates of the centrioles A and B were determined with Fiji. The direction of cell divisions was represented by the orientation of a vector $\overrightarrow{AB}$ between the centrioles. Correction of the anterior-posterior axis of each transversal section according to the embryonic anatomy was performed similarly as described for cell orientation.

Supplementary References

[1] B. Schmid, J. Schindelin, A. Cardona, M. Longair, and M. Heisenberg, 'A high-level 3D visualization API for Java and ImageJ.', *BMC Bioinformatics*, vol. 11, p. 274, May 2010, doi: 10.1186/1471-2105-11-274.

[2] M. Steinberg, 'A non-nutrient culture medium for amphibian embryonic tissues.', in *Carnegie Inst. Wash. Yearbook*, vol. 56, 1957, pp. 347–348.

[3] J. A. Dent, A. G. Polson, and M. W. Klymkowsky, 'A whole-mount immunocytochemical analysis of the expression of the intermediate filament protein vimentin in Xenopus.', *Development*, vol. 105, no. 1, pp. 61–74, Jan. 1989, doi: 10.1242/dev.105.1.61.

[4] L. McHedlishvili *et al.*, 'Reconstitution of the central and peripheral nervous system during salamander tail regeneration.', *Proc Natl Acad Sci U S A*, vol. 109, no. 34, pp. E2258-2266, Aug. 2012, doi: 10.1073/pnas.1116738109.

[5] T. Kurth, S. Weiche, D. Vorkel, S. Kretschmar, and A. Menge, 'Histology of plastic embedded amphibian embryos and larvae.', *Genesis*, vol. 50, no. 3, pp. 235–250, Mar. 2012, doi: 10.1002/dvg.20821.

[6] Y. Taniguchi *et al.*, 'The posterior neural plate in axolotl gives rise to neural tube or turns anteriorly to form somites of the tail and posterior trunk.', *Dev Biol*, vol. 422, no. 2, pp. 155–170, Feb. 2017, doi: 10.1016/j.ydbio.2016.12.023.

[7] S. Schneider, H. Steinbeisser, R. M. Warga, and P. Hausen, 'Beta-catenin translocation into nuclei demarcates the dorsalizing centers in frog and fish embryos.', *Mech Dev*, vol. 57, no. 2, pp. 191–198, Jul. 1996, doi: 10.1016/0925-4773(96)00546-1.

[8] A. Pérez Saturnino, K. Lust, and J. Wittbrodt, 'Notch signalling patterns retinal composition by regulating atoh7 during post-embryonic growth.', *Development*, vol. 145, no. 21, Nov. 2018, doi: 10.1242/dev.169698.

[9] W. Masselink *et al.*, 'Broad applicability of a streamlined ethyl cinnamate-based clearing procedure.', *Development*, vol. 146, no. 3, Feb. 2019, doi: 10.1242/dev.166884.

[10] M.-T. Ke, S. Fujimoto, and T. Imai, 'SeeDB: a simple and morphology-preserving optical clearing agent for neuronal circuit reconstruction.', *Nat Neurosci*, vol. 16, no. 8, pp. 1154–1161, Aug. 2013, doi: 10.1038/nn.3447.

[11] J. Schindelin *et al.*, 'Fiji: an open-source platform for biological-image analysis.', *Nat Methods*, vol. 9, no. 7, pp. 676–682, Jun. 2012, doi: 10.1038/nmeth.2019.

[12] J. Ollion, J. Cochennec, F. Loll, C. Escudé, and T. Boudier, 'TANGO: a generic tool for high-throughput 3D image analysis for studying nuclear organization.', *Bioinformatics*, vol. 29, no. 14, pp. 1840–1841, Jul. 2013, doi: 10.1093/bioinformatics/btt276.

Figure S1

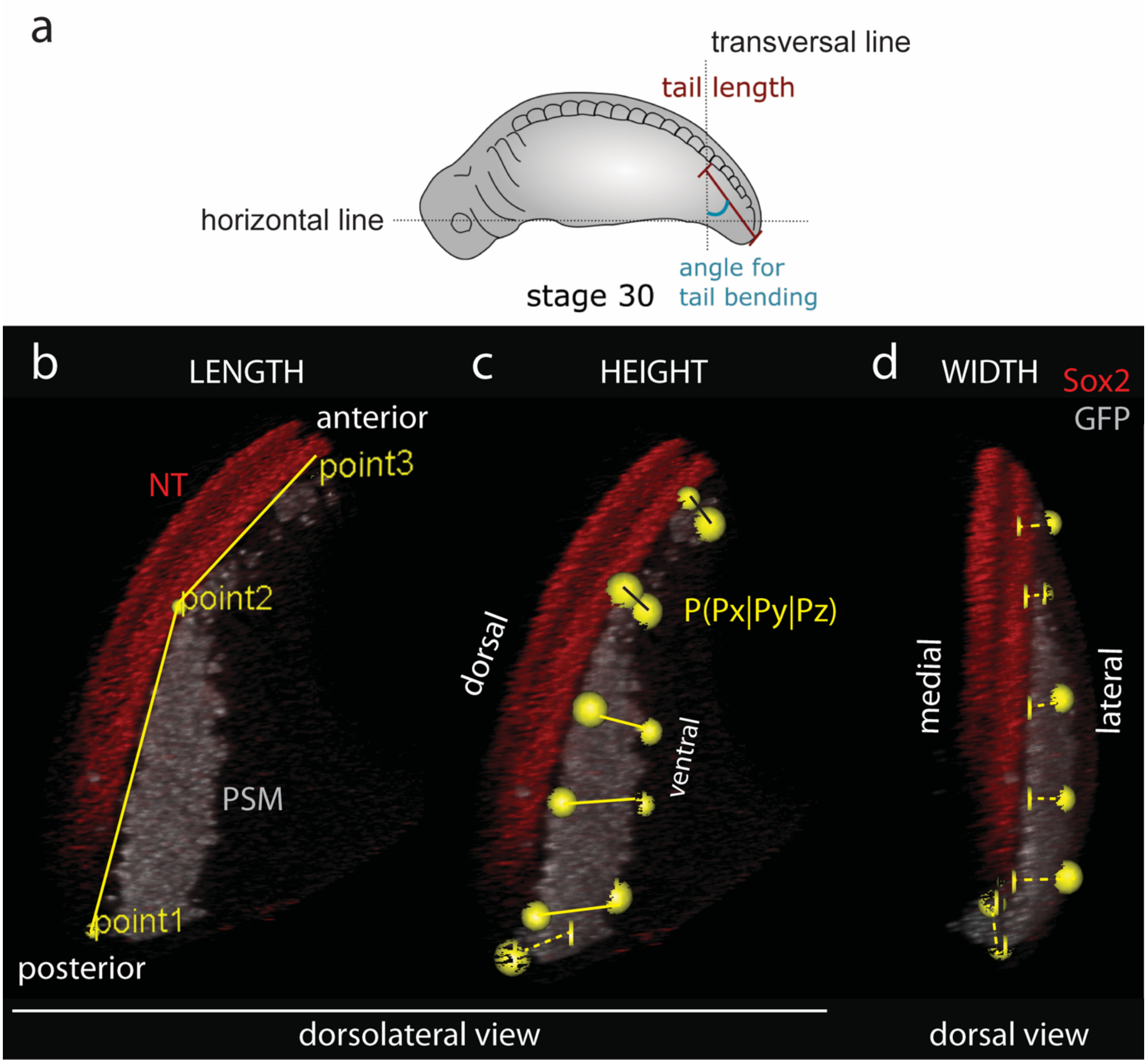
a
transversal line
tail length
horizontal line
angle for
tail bending
stage 30
b
LENGTH
anterior
point3
NT
point2
PSM
point1
posterior
c
HEIGHT
P(Px|Py|Pz)
dorsal
ventral
d
WIDTH
Sox2
GFP
medial
lateral
dorsolateral view
dorsal view

Figure S2

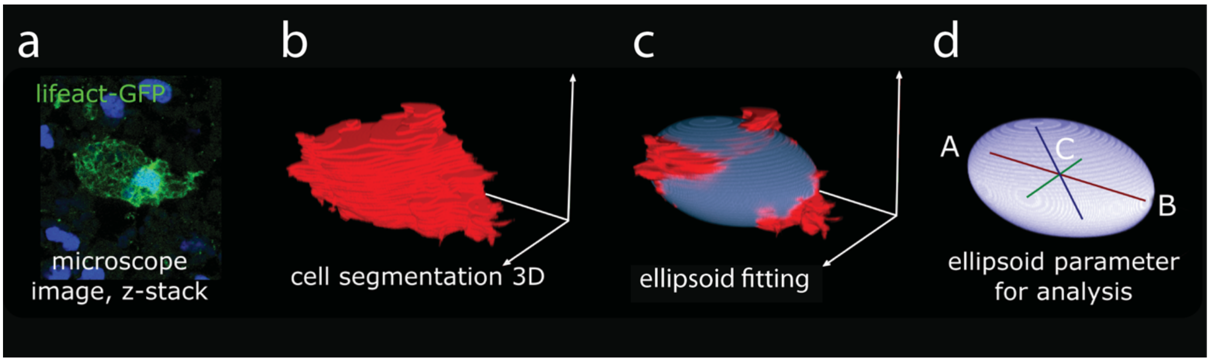

Figure S3

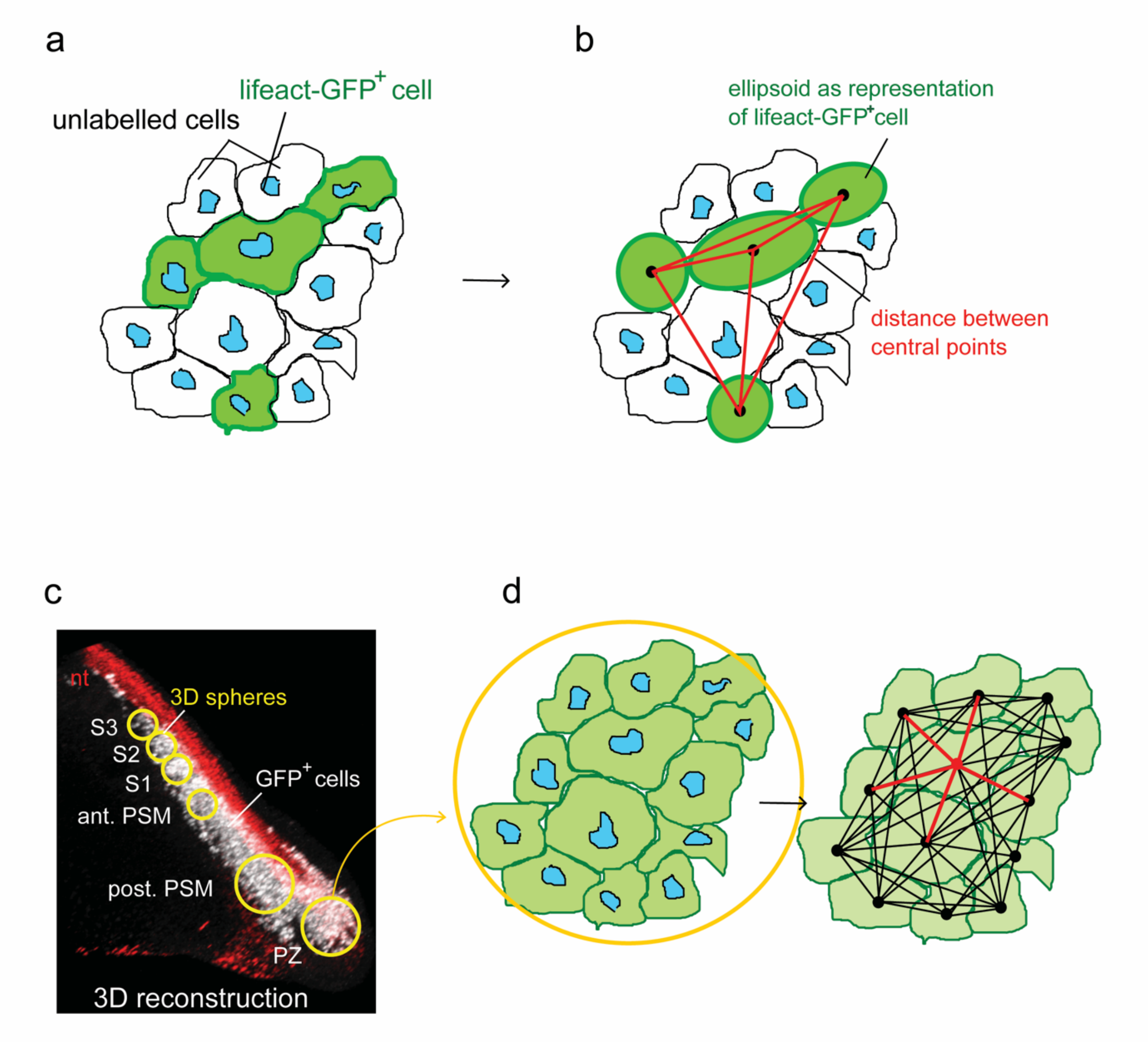

Figure S4

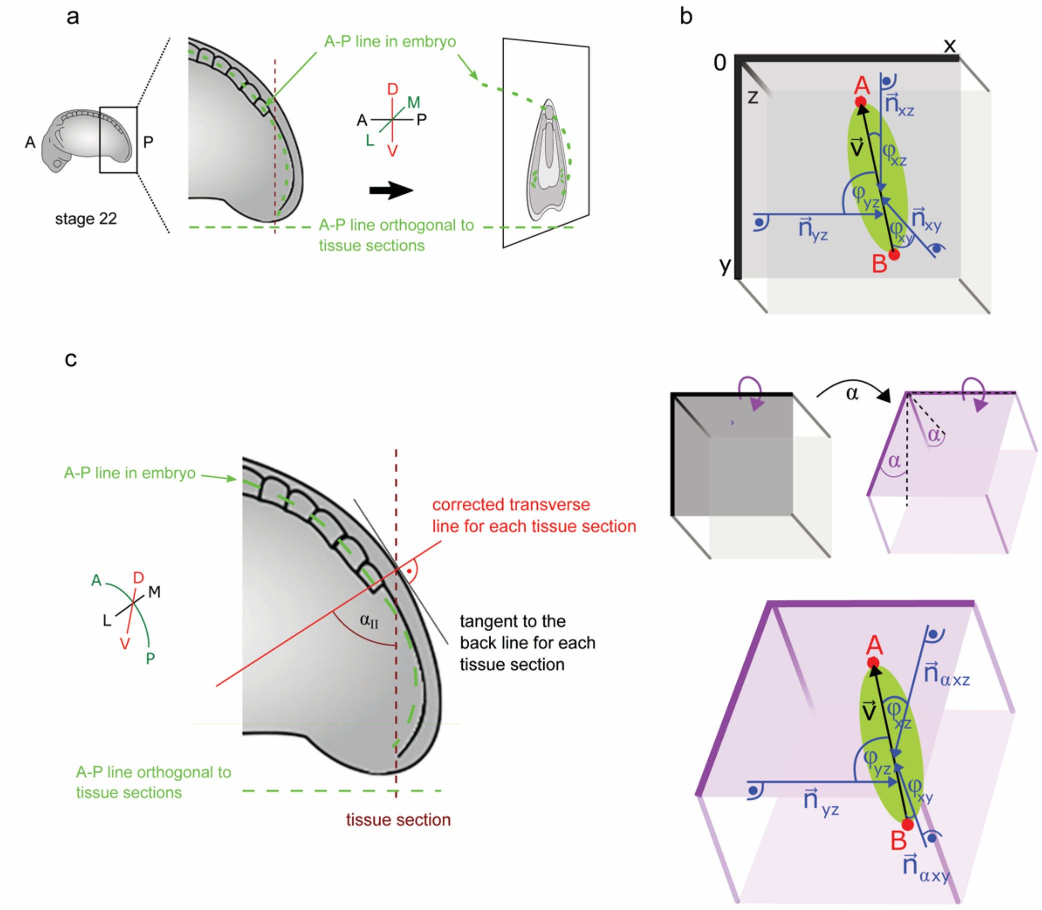

d

$$\vec{n}_{\alpha_{xz}} = \begin{pmatrix}1 & 0 & 0\\ 0 & \cos\alpha & -\sin\alpha\\ 0 & \sin\alpha & \cos\alpha\end{pmatrix} * \begin{pmatrix}0\\ 1\\ 0\end{pmatrix} = \begin{pmatrix}0\\ \cos\alpha\\ \sin\alpha\end{pmatrix}$$

$$\vec{n}_{\alpha_{xy}} = \begin{pmatrix}1 & 0 & 0\\ 0 & \cos\alpha & -\sin\alpha\\ 0 & \sin\alpha & \cos\alpha\end{pmatrix} * \begin{pmatrix}0\\ 0\\ 1\end{pmatrix} = \begin{pmatrix}0\\ -\sin\alpha\\ \cos\alpha\end{pmatrix}$$

Figure S5

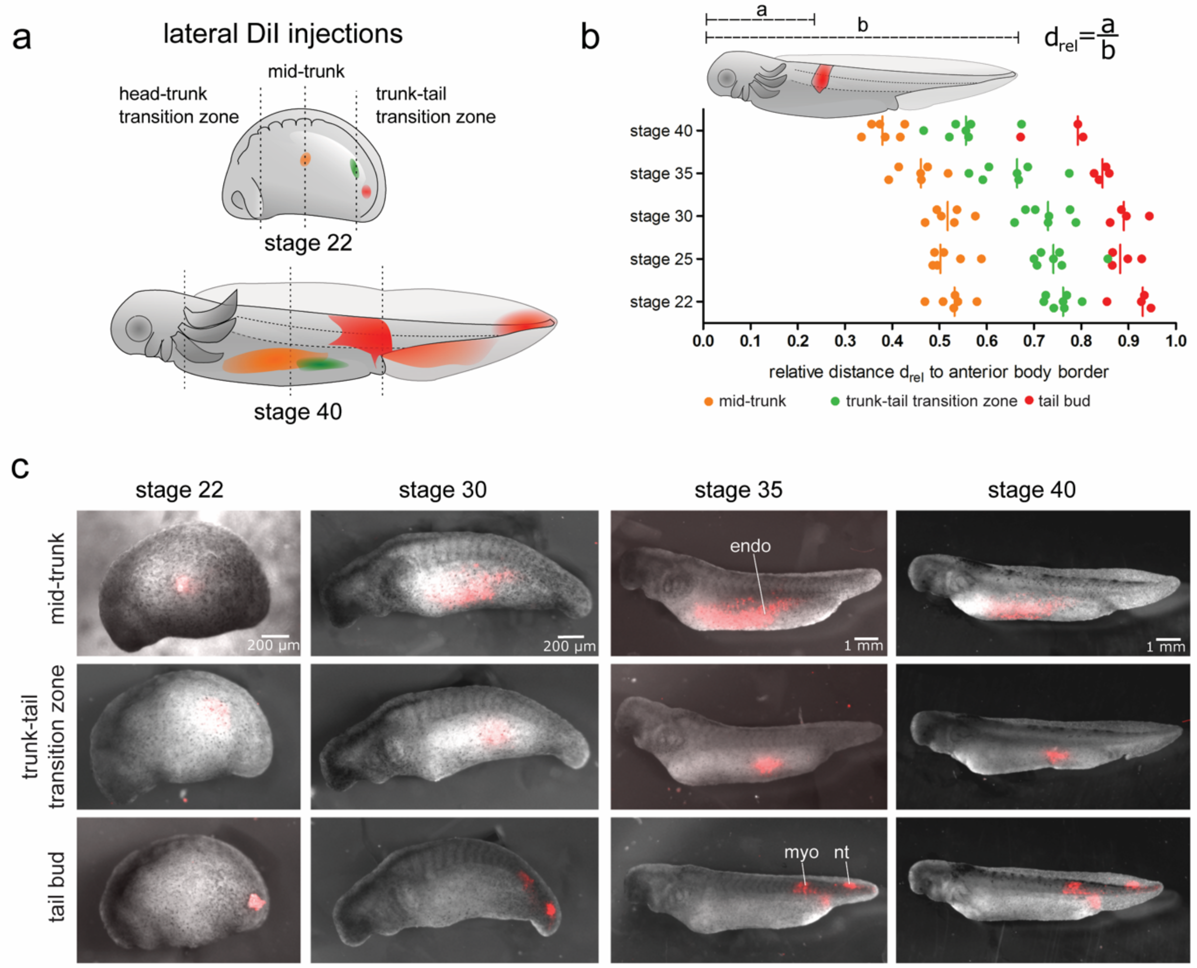
a
lateral DiI injections
mid-trunk
head-trunk transition zone
trunk-tail transition zone
stage 22
stage 40
b
a
b
$d_{rel}=\frac{a}{b}$
stage 40
stage 35
stage 30
stage 25
stage 22
0.0 0.1 0.2 0.3 0.4 0.5 0.6 0.7 0.8 0.9 1.0
relative distance d_{rel} to anterior body border
mid-trunk
trunk-tail transition zone
tail bud
c
stage 22
stage 30
stage 35
stage 40
mid-trunk
trunk-tail transition zone
tail bud
endo
myo
nt
200 µm
200 µm
1 mm
1 mm

Figure S6

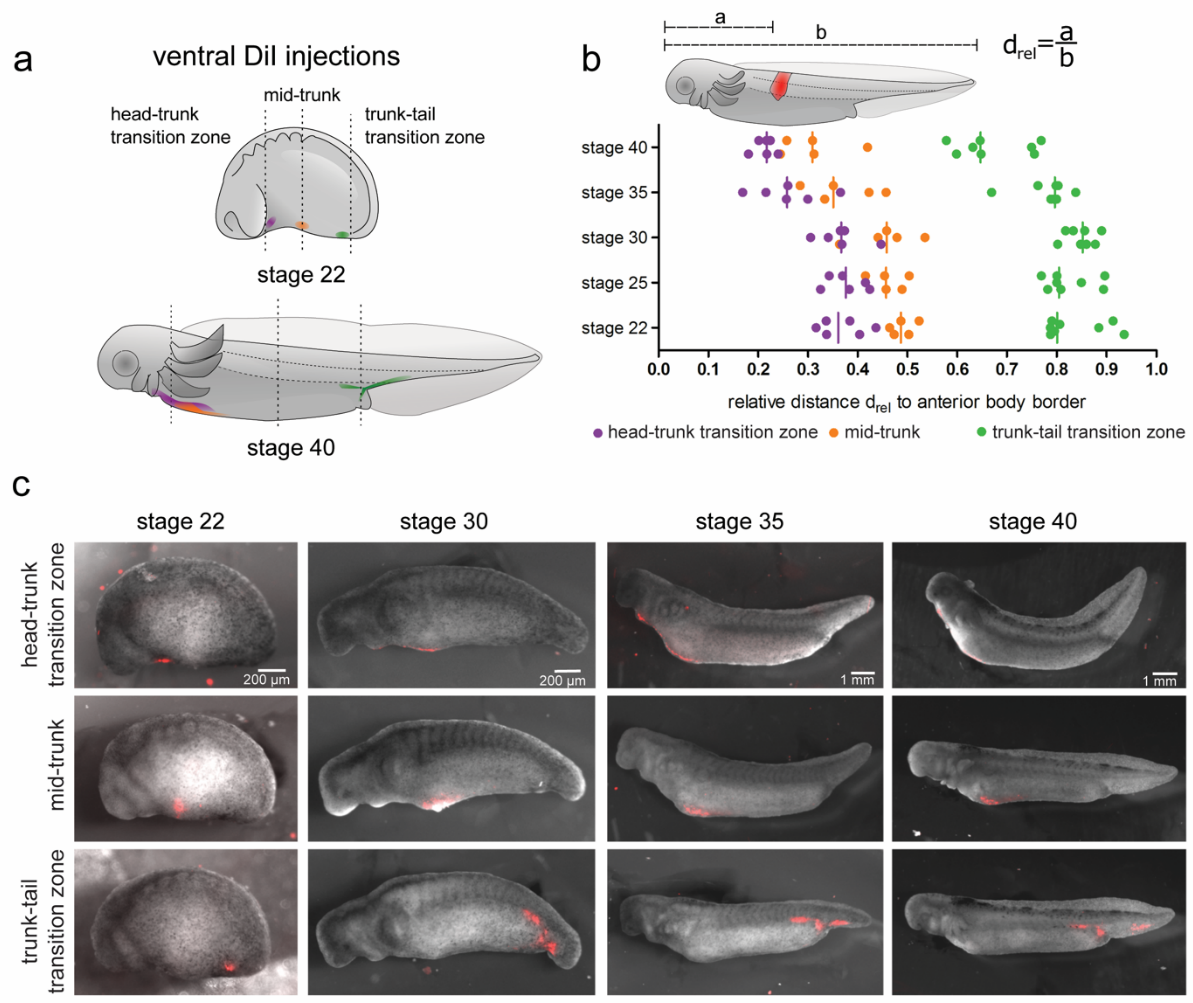
a
ventral DiI injections
head-trunk
transition zone
mid-trunk
trunk-tail
transition zone
stage 22
stage 40
b
a
b
d_rel = a/b
stage 40
stage 35
stage 30
stage 25
stage 22
0.0 0.1 0.2 0.3 0.4 0.5 0.6 0.7 0.8 0.9 1.0
relative distance d_rel to anterior body border
head-trunk transition zone
mid-trunk
trunk-tail transition zone
c
stage 22
stage 30
stage 35
stage 40
head-trunk transition zone
mid-trunk
trunk-tail transition zone
200 µm
200 µm
1 mm
1 mm

Figure S7

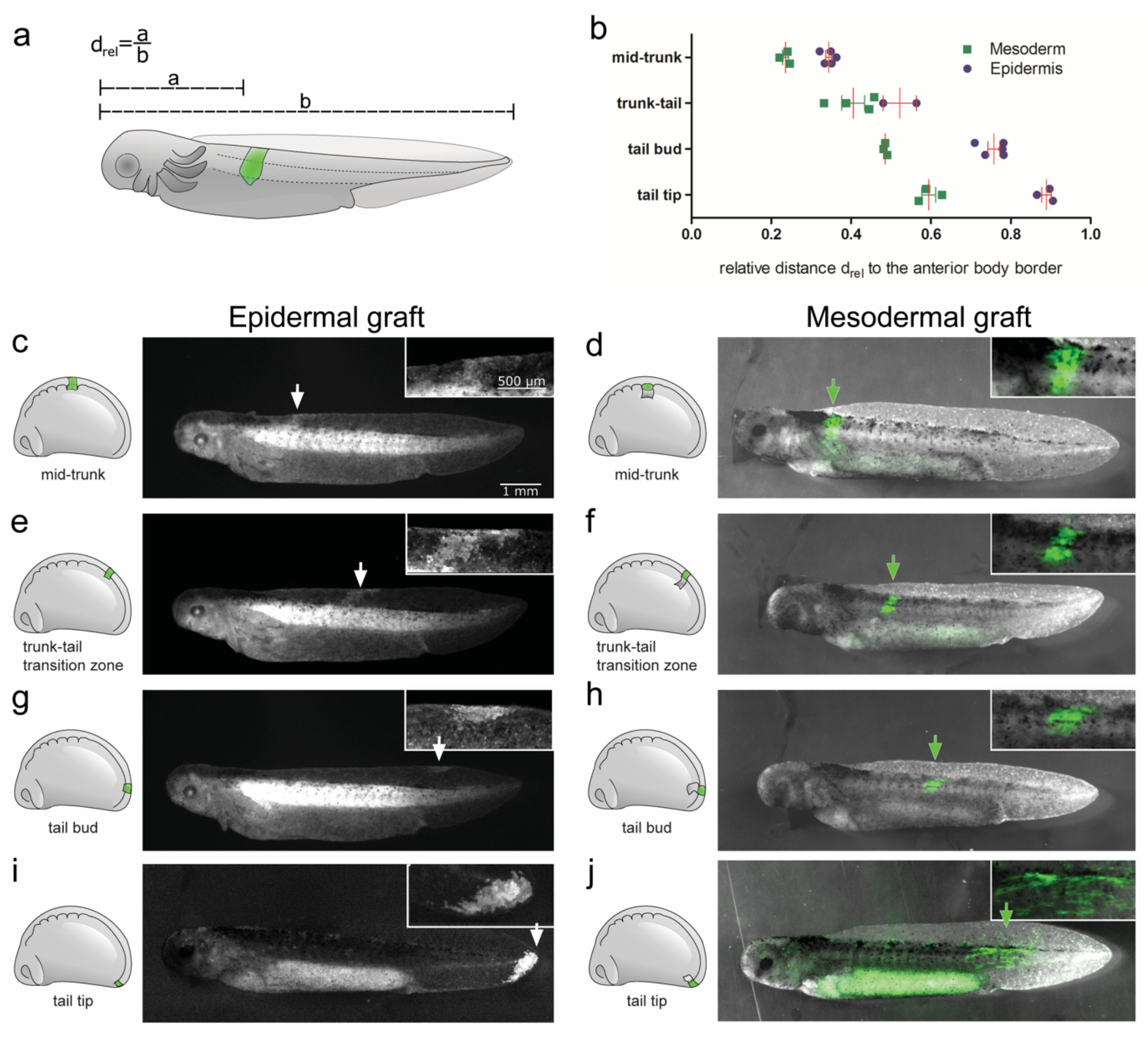
a
$d_{rel}=\frac{a}{b}$
a
b
b
mid-trunk
trunk-tail
tail bud
tail tip
Mesoderm
Epidermis
0.0
0.2
0.4
0.6
0.8
1.0
relative distance d_{rel} to the anterior body border
Epidermal graft
Mesodermal graft
c
500 µm
1 mm
mid-trunk
d
mid-trunk
e
trunk-tail
transition zone
f
trunk-tail
transition zone
g
tail bud
h
tail bud
i
tail tip
j
tail tip

Figure S8

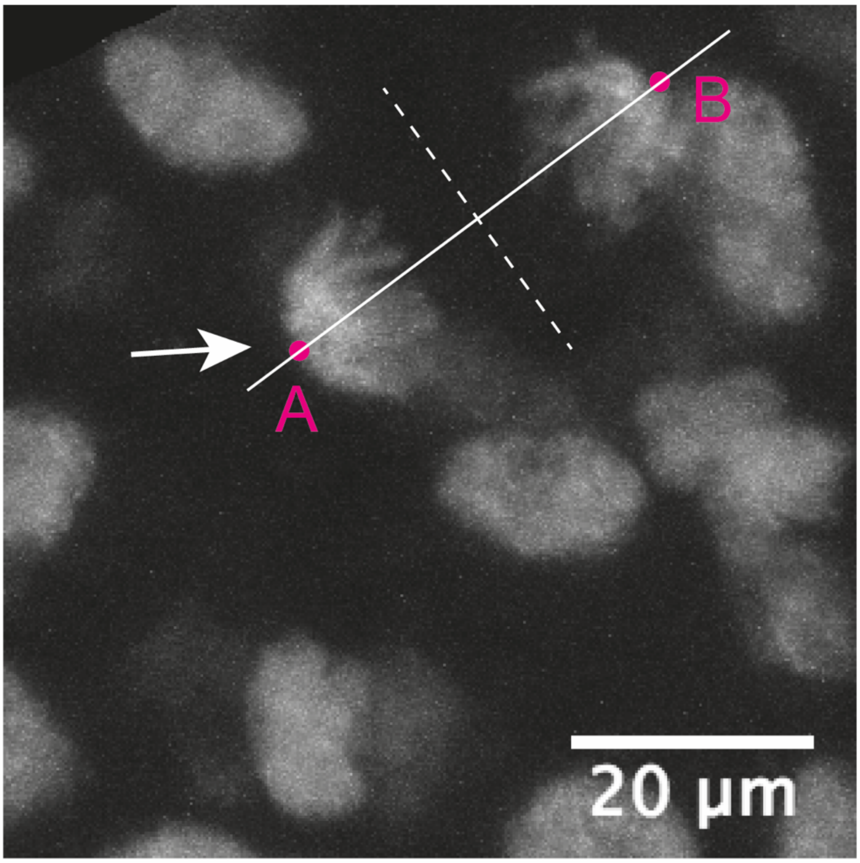

Figure S9

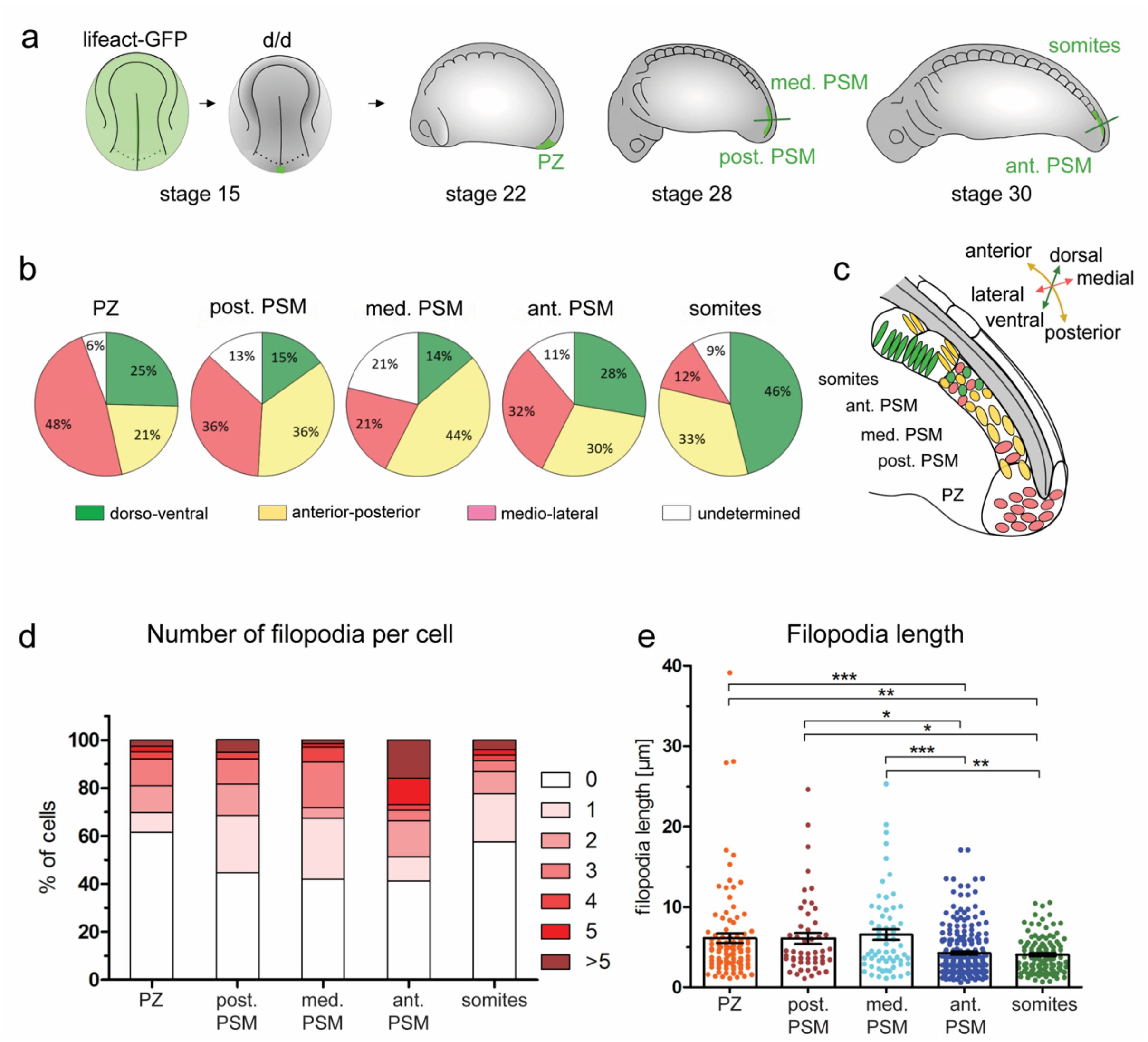

a
lifeact-GFP
d/d
stage 15
PZ
stage 22
med. PSM
post. PSM
stage 28
somites
ant. PSM
stage 30
b
PZ
post. PSM
med. PSM
ant. PSM
somites
6%
25%
48%
21%
13%
15%
36%
36%
21%
14%
21%
44%
11%
28%
32%
30%
9%
12%
46%
33%
dorso-ventral
anterior-posterior
medio-lateral
undetermined
c
anterior
dorsal
medial
lateral
ventral
posterior
somites
ant. PSM
med. PSM
post. PSM
PZ
d
Number of filopodia per cell
% of cells
0
1
2
3
4
5
>5
PZ
post. PSM
med. PSM
ant. PSM
somites
e
Filopodia length
filopodia length [µm]
***
**
*
*
***
**
PZ
post. PSM
med. PSM
ant. PSM
somites

Figure S10

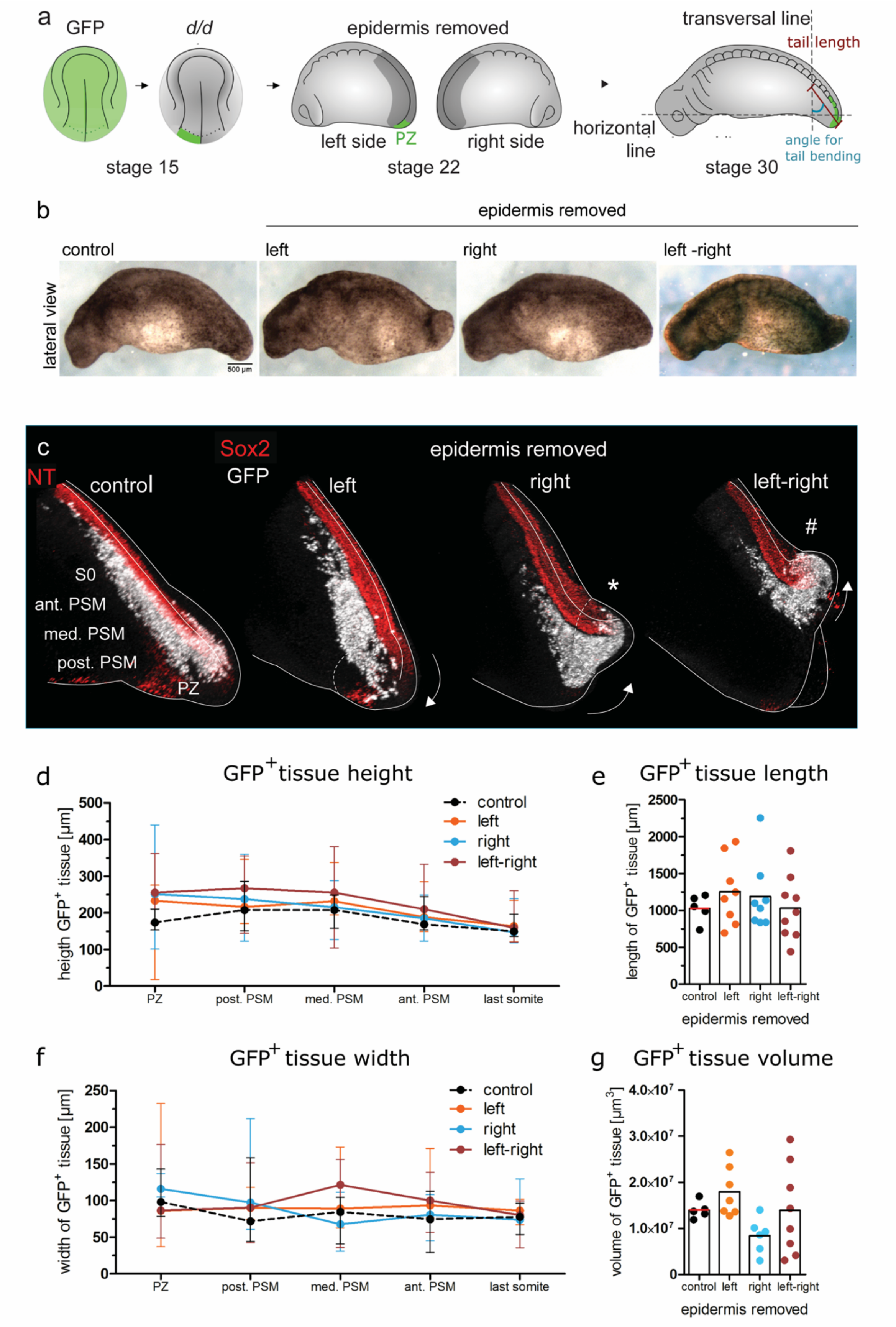

a
GFP
d/d
stage 15
epidermis removed
left side
PZ
right side
stage 22
transversal line
tail length
horizontal line
angle for tail bending
stage 30
b
epidermis removed
control
left
right
left -right
lateral view
500 µm
c
Sox2
GFP
NT
control
epidermis removed
left
right
left-right
S0
ant. PSM
med. PSM
post. PSM
PZ
*
#
d
GFP+ tissue height
heigth GFP+ tissue [µm]
control
left
right
left-right
PZ
post. PSM
med. PSM
ant. PSM
last somite
e
GFP+ tissue length
length of GFP+ tissue [µm]
control
left
right
left-right
epidermis removed
f
GFP+ tissue width
width of GFP+ tissue [µm]
g
GFP+ tissue volume
volume of GFP+ tissue [µm3]
4.0x10^7
3.0x10^7
2.0x10^7
1.0x10^7

Figure S11

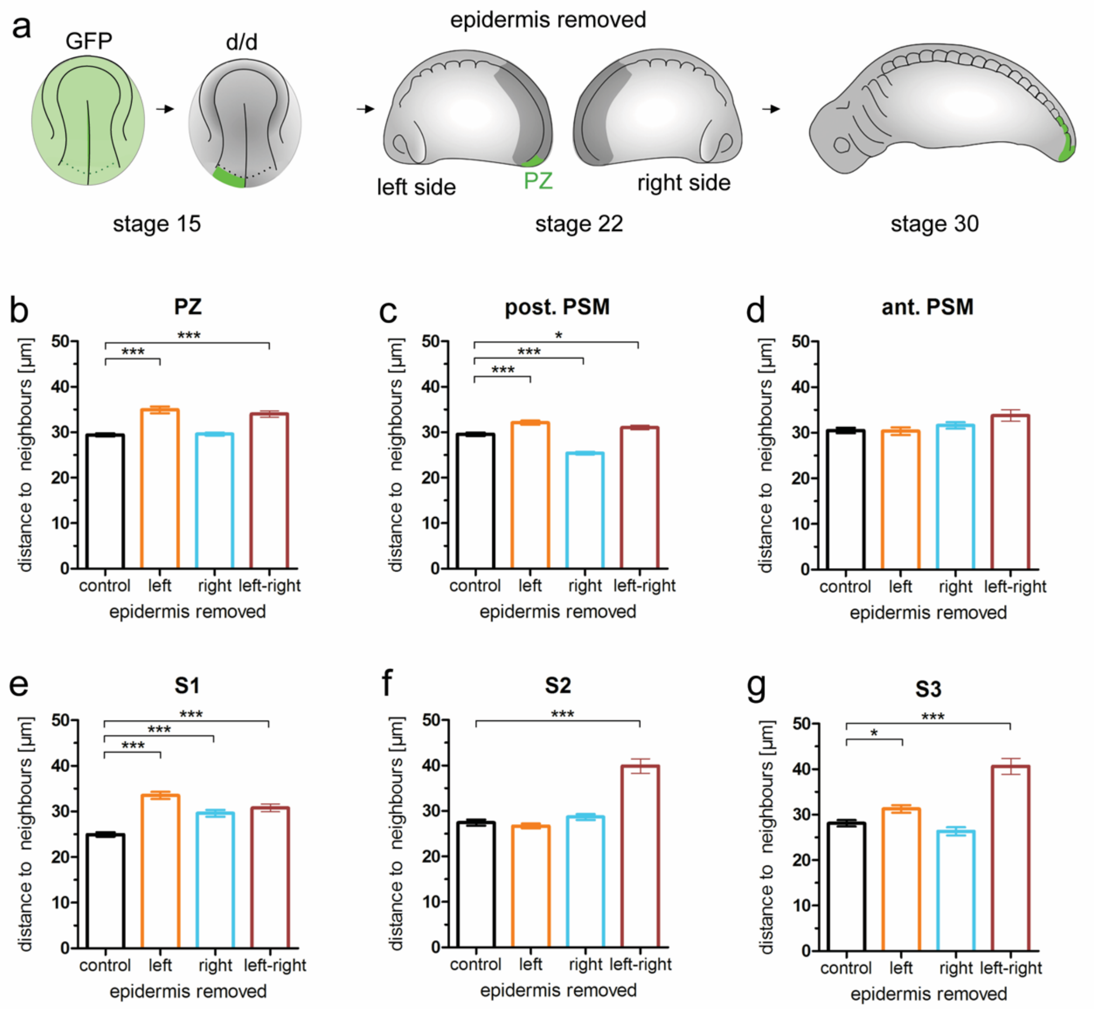
a
GFP
d/d
epidermis removed
left side
PZ
right side
stage 15
stage 22
stage 30
b
PZ
c
post. PSM
d
ant. PSM
e
S1
f
S2
g
S3
distance to neighbours [µm]
control
left
right
left-right
epidermis removed
***
*

Figure S12

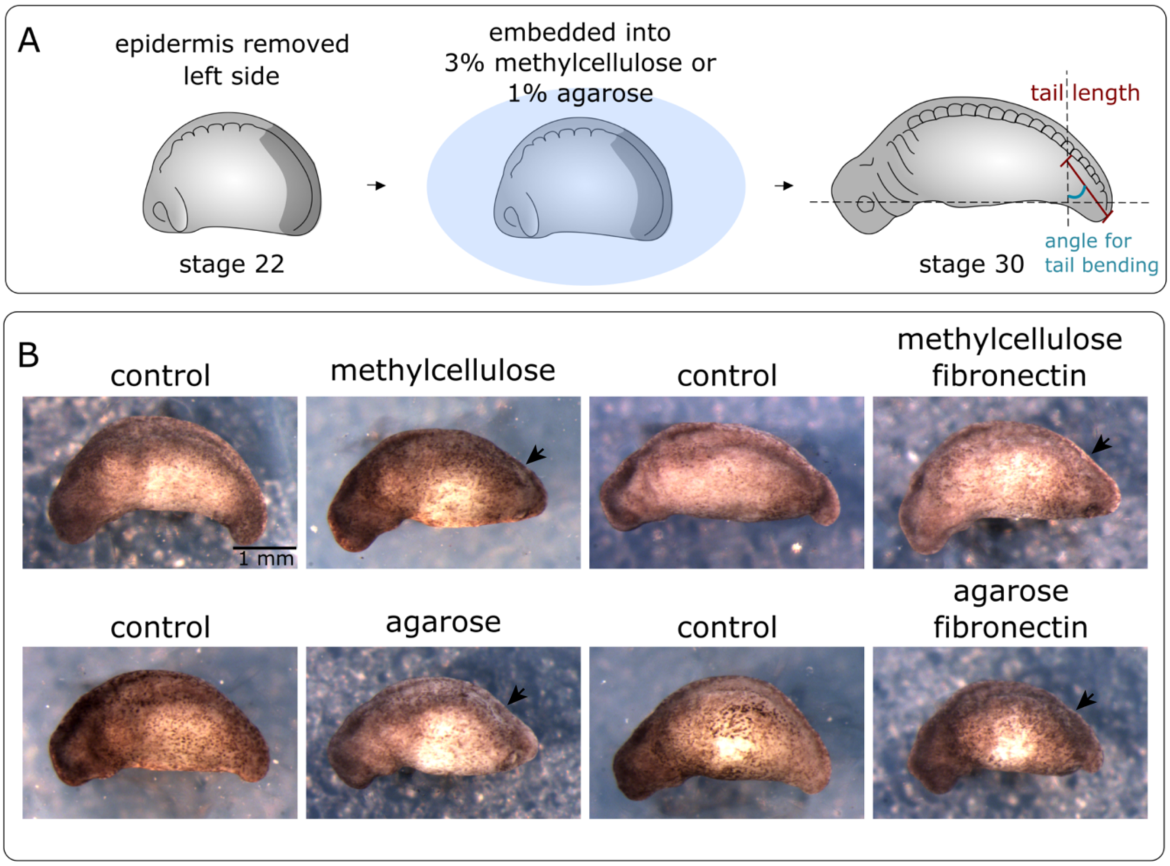
A
epidermis removed
left side
stage 22
embedded into
3% methylcellulose or
1% agarose
tail length
angle for
tail bending
stage 30
B
control
methylcellulose
control
methylcellulose
fibronectin
1 mm
control
agarose
control
agarose
fibronectin
